# Pulmonary Arterial Hypertension: Reconfiguring the Vascular Landscape to Reverse Remodeling

**DOI:** 10.3390/cells15040332

**Published:** 2026-02-11

**Authors:** Alice G. Vassiliou, Kostas A. Papavassiliou, Nikolaos S. Lotsios, Stylianos E. Orfanos, Athanasios G. Papavassiliou

**Affiliations:** 1 First Department of Critical Care Medicine, ‘Evangelismos’ Hospital, Medical School, National and Kapodistrian University of Athens, 10676 Athens, Greecestylianosorfanosuoa@gmail.com (S.E.O.); 2 First University Department of Respiratory Medicine, ‘Sotiria’ Chest Hospital, Medical School, National and Kapodistrian University of Athens, 11527 Athens, Greece; konpapav@med.uoa.gr; 3 Department of Biological Chemistry, Medical School, National and Kapodistrian University of Athens, 11527 Athens, Greece

The therapeutic interventions for pulmonary arterial hypertension (PAH) have predominantly focused on facilitating pulmonary blood flow by inducing vasodilation to reduce the mechanical resistance against the right ventricle (RV) [1]. However, the current pharmacological options, which include prostacyclin (also called prostaglandin I_2_ or PGI_2_) analogs, endothelin receptor (ETR) antagonists, and phosphodiesterase-5 (PDE5) inhibitors, insufficiently arrest the underlying pathological structural alterations leading to RV failure, which persists as the primary driver of mortality in patients with PAH [2]. The recent clinical introduction of sotatercept, an activin signaling inhibitor, represents a pivotal shift toward disease-modifying therapy that rebalances pro-proliferative and antiproliferative signaling [3]. Yet, although sotatercept addresses the transforming growth factor beta (TGFβ) family imbalance, it also highlights the profound complexity of the pulmonary vascular environment and the need for interventions that address the cause of cellular dysfunction. The central challenge in PAH research remains incomplete genetic penetrance. The most notable mutations in the *bone morphogenetic protein receptor type 2* (*BMPR2*) gene, which occur in approximately 70–80% of heritable PAH and 20% of sporadic cases or idiopathic PAH (IPAH), establish a primary risk foundation; however, they often do not manifest as clinical disease. This suggests that the transition from a predisposed state to pathology is governed by an epigenetic second hit. By framing PAH as a reversible epigenetic state, the scientific community can shift the therapeutic focus from mitigating symptoms to actively reversing vascular remodeling.

The “Double-Hit” model provides the most compelling explanation of PAH’s pathogenesis [4]. The “First Hit” is the inherited or de novo genetic foundation, creating a vulnerable vascular environment characterized by impaired growth-suppressive signaling. The low penetrance of these mutations (approximately 20%) proves that these fixed genomic sequences are not sufficient to trigger the catastrophic remodeling seen in clinical practice. The “Second Hit” is the epigenetic catalyst. The epigenome serves as a dynamic regulatory layer; in predisposed individuals, environmental triggers, such as chronic inflammation, localized hypoxia, viral insults, and oxidative stress, trigger a cascade of epigenetic modifications [5]. These are not genetic variants: they are chemical marks on the DNA and its packaging proteins, histones, that reprogram cellular behavior. Through DNA methylation, histone acetylation, and non-coding RNA interference, the pulmonary artery smooth muscle cells (PASMCs) and endothelial cells (ECs) transition from a quiescent, functional state into a hyper-proliferative, apoptosis-resistant phenotype, leading to vascular remodeling. Because these modifications are enzymatic rather than structural changes in the DNA sequence, they are theoretically reversible. This is the pivotal point for a new therapeutic paradigm: if we can erase the pathological “memory” of the second hit, we may return the cell to its original, healthy state.

To understand how to reverse remodel, we must first understand the specific epigenetic marks that maintain the diseased state. High levels of DNA methyltransferase (DNMT) activity can lead to the hypermethylation of the promoter regions for genes essential to vascular health. A prime example is the silencing of *superoxide dismutase 2* (*SOD2*), which impairs mitochondrial function and pushes cells toward a glycolytic, “Warburg-like” metabolism that favors rapid growth over specialized function [6]. Ten-eleven translocation (TET) enzymes, specifically TET2, are the key enzymes in DNA demethylation. They are found in cardiovascular disease and associated with clonal hematopoiesis, inflammation, and adverse vascular remodeling. Investigations have demonstrated that *TET2* expression is significantly downregulated in the pulmonary vasculature of patients with PAH and in experimental models [7]. Histone deacetylases (HDACs) actively remove acetyl groups from histones, causing the chromatin to coil tightly and silence gene expression. In PAH, specific Class I and IIa HDACs are upregulated, silencing the potassium channels and pro-apoptotic factors required to maintain normal vascular growth [8]. Other HDACs, sirtuins (SIRTs), have been also implicated in PAH. Sirtuins regulate important metabolic pathways in biological processes including cell survival, proliferation, apoptosis, DNA repair, and cell metabolism. Mice lacking SIRT3, a mitochondrial deacetylase, exhibited suppressed mitochondrial oxidative metabolism and spontaneously developed PH. Furthermore, a loss-of-function *SIRT3* polymorphism has been linked to PAH development in humans [9]. Histone acetylation serves as a fundamental regulatory mechanism of chromatin structure, creating docking sites for bromodomains. The epigenetic reader bromodomain-containing protein 4 (BRD4) is a transcriptional co-activator that binds to acetylated lysine residues on histone proteins and facilitates the recruitment of the transcriptional machinery to target genes. BRD4 is often overexpressed in the lungs of those with PAH, where it recruits transcription factors that drive the expression of pro-survival proteins such as survivin and B-cell lymphoma 2 (Bcl-2) [10]. In addition to the direct enzymatic modification of DNA and histones, the epigenetic landscape of PAH is heavily influenced by non-coding RNAs, particularly microRNAs (miRNAs). These small RNA molecules act as post-transcriptional regulators, often silencing genes that maintain vascular stability. In the lung of those with PAH, a specific “miRNA signature” emerges, characterized by the downregulation of anti-proliferative species such as miR-204 and miR-124, and the upregulation of pro-remodeling species such as miR-21 [11,12,13,14]. This imbalance seems to further drive the hyper-proliferative phenotype of PASMCs and ECs. The potential therapeutics “antagomirs” (inhibitors of pathological miRNAs) or miRNA mimics (to restore protective signaling) offer a high degree of specificity, potentially allowing for the targeted reversal of the molecular pathways that bypass traditional genetic control. By targeting these RNA-mediated pathways, researchers are exploring “antagomirs” and mRNA-based restoration therapies to reset the vascular cell’s regulatory environment [15].

The shift toward viewing PAH as an epigenetic disease is already manifesting in the clinical trial pipeline. The identification of these “switches” has led to the development of a new class of agents known as “epidrugs”. These small molecules are designed to target the switches of the epigenetic code and reset the vascular landscape. The use of HDAC inhibitors in treating PAH is perhaps the most advanced epigenetic strategy. By inhibiting these enzymes, we can prevent the silencing of homeostatic genes. Studies explored the use of valproic acid (VPA) and more selective Class I HDAC inhibitors [16]. While early-stage clinical work is ongoing, the preclinical data are supportive, showing not just a slowing of disease but significant reductions in right ventricular hypertrophy (RVH) and fibrosis [17,18]. The challenge that remains is the systemic toxicity of non-selective HDAC inhibition, leading to a push for lung-targeted delivery systems [19]. Dichloroacetate (DCA) is a pyruvate dehydrogenase kinase (PDK) inhibitor that promotes glucose oxidation, and its recent clinical trial results underscore the importance of the metabolism−epigenetics axis. The study demonstrated improvements in PAH in genetically susceptible patients [20]. Targeting readers such as BRD4 offers a method of interrupting the massive inflammatory and proliferative signaling characteristic of late-stage PAH [10,21,22,23]. Apabetalone, a bromodomain and extra-terminal motif (BET) inhibitor originally studied for cardiovascular disease, produced improvements in pulmonary vascular resistance (PVR) and other hemodynamic parameters in a pilot clinical trial [24]. By blocking BRD4, the drug effectively silences the pathological genes that the epigenetic second hit induced. If DNA methylation silences protective genes, DNMT inhibitors activates them. Low-dose decitabine, a drug traditionally used in hematological malignancies, is being repurposed for PAH. Research suggests that, at low doses, it can restore the expressions of *SOD2* and other silenced genes, reversing the metabolic switch that drives PASMC proliferation [6,25].

The scientific community must recognize that we are at crossroads. The “Double-Hit” model provides us with a clear roadmap: we cannot easily change the genetic foundation (the first hit), but we can certainly address the epigenetic marks (the second hit). However, to advance these approaches into clinical trials, we must overcome three critical challenges. The first is tissue specificity; since epigenetic marks exist in every cell, developing inhaled formulations or ligand-targeted nanoparticles that deliver epidrugs directly to the pulmonary arteries is essential to avoid systemic side effects. Second, we need to determine if epigenetic reversal is possible in plexiform lesions or if these therapies are most effective in the early proliferative stages of remodeling. Finally, we need non-invasive methods for measuring the epigenetic load of a patient. Profiling circulating microRNAs or DNA methylation patterns could allow us to tailor epigenetic regimens to the individual’s specific “Second Hit”.

Research evidence is increasingly supporting a shift in how we conceptualize PAH. It is not merely an irreversible genetic fate but a condition maintained by a reversible epigenetic state. By utilizing agents that target these molecular drivers, we move beyond vasodilation. The objective now is to reconfigure the pulmonary vasculature, erasing the marks of the second hit, with the aim of restoring the functional integrity of the vascular bed and achieve true disease modification.

## Data Availability

No new data were generated in this work.
